# Dairy product intake and bone properties in 70-year-old men and women

**DOI:** 10.1007/s11657-018-0420-1

**Published:** 2018-01-29

**Authors:** Olle M. Hallkvist, Jonas Johansson, Anna Nordström, Peter Nordström, Andreas Hult

**Affiliations:** 1 Department of Public Health and Clinical Medicine, Section for Occupational and Environmental Medicine, Umeå University, SE-90187 Umeå, Sweden; 2 Department of Community Medicine and Rehabilitation, Geriatric Medicine, Umeå University, Umeå, Sweden

**Keywords:** Dairy product, Bone mineral density, Peripheral quantitative computed tomography, Older adults

## Abstract

**Summary:**

In the present population-based study including 70-year-old men and women, total dairy product intake was associated with a weak positive association with tibia trabecular and cortical cross-sectional areas.

**Purpose:**

Milk consumption has recently been suggested to increase fracture risk. Therefore, we aimed to investigate associations between dairy product consumption and peripheral bone properties. Furthermore, we explored whether consumption of milk and fermented dairy products affected bone properties differently.

**Methods:**

The Healthy Aging Initiative is a population-based, cross-sectional study investigating the health of 70-year-old men and women. Out of the 2904 individuals who met the inclusion criteria, data on self-reported daily dairy product consumption (dl/day), peripheral quantitative computed tomography (pQCT) examinations at the 4 and 66% scan sites of the tibia and radius, and dual-energy X-ray absorptiometry (DXA) scans were collected from 2040 participants. Associations between dairy product consumption and bone properties were examined using multiple linear regression models adjusted for sex, muscle area, meal size, dietary protein proportion, current smoking status, and objectively measured physical activity.

**Results:**

Total dairy product intake was associated with larger trabecular (2.296 (95% CI, 0.552–4.039) mm^2^, per dl/day increase, *p* = 0.01) and cortical cross-sectional areas (CSAs) in the tibia (1.757 (95% CI, 0.683–2.830 mm^2^, *p* = 0.001) as measured by pQCT and higher areal bone mineral density (aBMD) of the radius (3.231 (95% CI, 0.764–5.698) mg/cm^2^, *p* = 0.01) as measured by DXA. No other measurement in the tibia, radius, femoral neck, or lower spine was associated significantly with dairy product intake. Bone properties did not differ according to the type of dairy product consumed.

**Conclusion:**

No evidence of a negative association between dairy product consumption and bone health was found. Furthermore, total dairy product consumption was associated with increased CSAs in the tibia, regardless of dairy product type. Collectively, our findings indicate the existence of a weak but significant positive association between dairy product consumption bone properties in older adults.

## Introduction

The number of elderly adults in the population is increasing steadily as a consequence of the economic, social, medical, and technological improvements of the past century. By 2050, projections suggest that there will be more old than young persons for the first time in history [1]. Aging is associated with the deterioration of physiological capacity, including poorer bone health [2]. The body constantly remodels bone to repair breaks and other damage and to strengthen the skeleton in the presence of greater mechanical loads [3]. Bone mass peaks after the second decade of life [4] and then begins to decline [2, 5], resulting in frailty in some individuals over time, which, in turn, is associated with an increased risk of fracture [4].

Several intervention studies have been conducted with the aim of reducing the incidence of age-related frailty fractures. Increased dietary calcium intake, particularly the intake of dairy products (which contain large amounts of calcium [6]), has been investigated, although evidence for its positive effects on bone health and fracture risk has been inconclusive [4, 7–9], and it has even been associated with increased fracture risk and mortality [10].

Most studies investigating the effect of dairy product intake on bone health have used dual-energy X-ray absorptiometry (DXA) to measure bone properties [9, 11]. These studies have thus been limited to two-dimensional measurements of areal bone mineral density (aBMD) and bone mineral content. Peripheral quantitative computed tomography (pQCT) enables volumetric (v) measurement of peripheral bone properties (including vBMD) and the distinction between cortical and trabecular bone [12], otherwise unattainable in the standard aBMD quantification by DXA. Few studies investigating the effects of dairy product intake, however, have used pQCT for bone measurement [13, 14].

The primary aims of this study were to investigate the associations between dairy product intake and bone properties measured by DXA and pQCT and to determine whether these relationships differed between the upper and lower extremities, in men and women aged 70 years. A secondary aim of the study was to investigate whether bone properties differed depending on the type of dairy product consumed.

## Materials and methods

### Study population

**Fig. 1:**
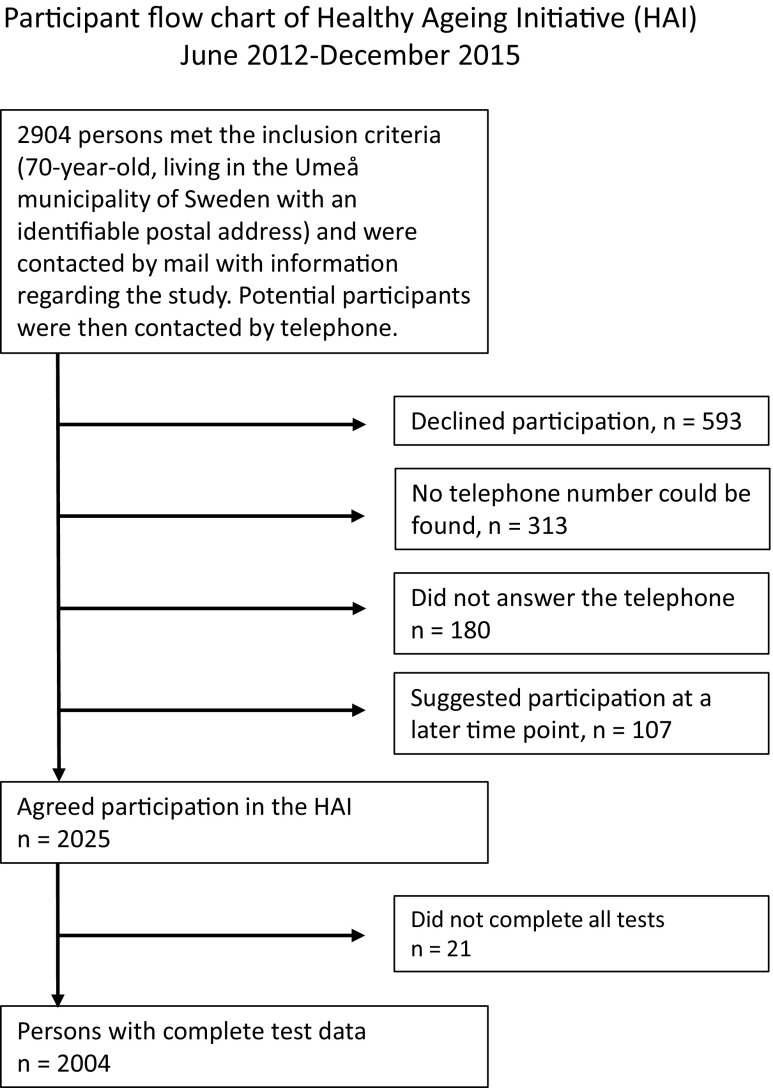
Participant flow chart of individuals meeting the inclusion criteria of the Healthy Aging Initiative study between June 2012 and December 2015

The Healthy Aging Initiative (HAI) is an ongoing population-based study with the aim of investigating traditional and new potential risk factors for cardiovascular disease, injurious falls, and related fractures among 70-year-old men and women. Between June 2012 and December 2015, 2904 individuals met the inclusion criteria: 70 years old and living in the Umeå municipality of northern Sweden (latitude 64° N) with an identifiable postal address. In the present cross-sectional study, we analyzed data collected from the first 2040 HAI participants (51.0% men) for whom complete pQCT bone measurements were available. All potentially eligible individuals were sent written information about the study using contact information from population registers. These individuals were then contacted by telephone and invited to participate. The participation rate of the HAI study, calculated as the percentage of individuals who had received the initial information and then completed testing, was 69%. Those who had not participated when this compilation was made were not contactable, planned to participate at a later date, or declined participation upon contact by telephone (see Fig. 1 for participation flow chart).

### Measurements

#### Anthropometrics

Body height (m) was determined with a height-measuring gauge (Holtain Limited, Crymych, Dyfed, Britain). Body weight (kg) was determined using a scale (HL 120; Avery Berkel, Smethwick, UK). Body mass index was calculated as weight divided by height squared. Waist and hip circumferences (in centimeters) were measured using a measuring tape. Experienced research nurses performed all anthropometric measurements.

#### pQCT

Bone properties were measured with a pQCT device (XCT-2000; Stratec Medizintechnik, Pforzheim, Germany). The following measurements were taken on participants’ non-dominant tibiae and radii: trabecular and cortical cross-sectional areas (CSAs, mm^2^), vBMD (mg/cm^3^), and cortical thickness (mm). During each examination, a digital scout view was first acquired to visualize the joint space, and a reference point was set electronically at the distal end of each bone (tibia and radius) as described previously [2]. The vBMD and CSA of trabecular bone were measured at scan sites located at 4% of the total bone length in the distal-proximal direction. The vBMD, CSA, and thickness of cortical bone were measured at scan sites located at 66% of the total bone length in the same direction. The scans had a slice thickness of 2.0 mm and a voxel size of 0.5 mm. Thresholds for analysis were set at 180 mg/cm^3^ for trabecular scans and 280 mg/cm^3^ for cortical scans. When motion artifacts were detected, measurements were repeated. In vivo coefficients of variance (CVs) for the Stratec XCT-2000 pQCT device are 1.6% for trabecular density and 0.3% for cortical density [15]. Three technicians performed the scans using the same machine.

#### iDXA

aBMD (g/cm^2^) was measured in the left femoral neck, lumbar spine (L1–L4), and the left radius using a Lunar iDXA device (GE Healthcare Lunar, Madison, WI, USA). The aBMD of the radius was measured at 33% of the total bone length in the distal-proximal direction. Standard DXA units of aBMD were transformed (g/cm^2^ to mg/cm^2^) in order to facilitate comparison between iDXA data and pQCT data. The coefficient of variation (standard deviation/mean) for repeated measurements at our facility is less than 2% for all iDXA measurements.

#### Food frequency questionnaire

The participants completed a food frequency questionnaire (FFQ) used to evaluate their current usual food habits. Daily liquid dairy product consumption was assessed by estimation of the volume of dairy products consumed (dl/day). Calcium- and vitamin D-enriched soy/oat/rice beverages were included in this category, in addition to milk and liquid-fermented dairy products. Additional questions concerning dairy intake included a question whether the liquid dairy intake consisted of milk, fermented dairy, both or neither, and a question assessing cheese consumption by asking how many slices of cheese the participants ate per day. Other included questions relevant for the study assessed estimated total meal size (5 grade scale from less than one half portion size to large portion size) and estimation of serving size of protein (represented as meat/fish) from photos showing one half portion size, three fourths portion size, normal portion size, and large portion size. The questionnaire also contained questions about participants’ tobacco habits. The participants were asked to fill out the questionnaire to the best of their abilities and on their own, unless they had questions. FFQ responses were recoded and used to evaluate total dairy product intake and types of dairy product consumed. Participants were assigned to the following four groups: milk only (MG), fermented dairy products only (FG), all dairy products (both milk and fermented dairy, ADG), and no milk or fermented dairy product (NDG).

#### Objective measurement of physical activity

At the end of the testing session, each participant was asked to wear a triaxial accelerometer (GT3X+; ActiGraph, Pensacola, FL, USA) for seven consecutive days, as described previously [16]. When participants returned with the accelerometers, the data were downloaded and wear time was validated using the ActiLife software (version 6.11.2; ActiGraph). Participants were required to accumulate at least 10 h of physical activity per day, for a total of 4 days, to be eligible for further analysis. Physical activity of 100–1951 counts per minute (CPM) was classified as light, and ≥ 1952 CPM was classified as moderate to very vigorous, in accordance with previous studies [17, 18]. Total counts from the three axes of the accelerometer were combined and divided by total wear time to generate a standardized measure of physical activity. The validity [19, 20] and reliability [21] of ActiGraph data have been tested thoroughly in previous studies.

### Ethics

The Umeå University Research Ethics Committee approved this study (Dnr 07-031M, with extensions). The study was conducted in accordance with the Declaration of Helsinki of the World Medical Association, and all participants provided written informed consent.

### Statistical analyses

Descriptive data were presented as means and standard deviations for continuous variables and percentages for categorical variables. Differences between men and women were investigated using independent *t* tests for continuous data and chi-squared tests for categorical data. Multiple linear regression models were used to examine associations of total intake of milk and fermented dairy products with bone properties. The first model was adjusted for sex, and the second model was additionally adjusted for sex, average portion size, dietary protein content, cross-sectional muscle area measured by pQCT, smoking, and physical activity. Potential associations between bone properties and total dairy product intake were presented using standardized *β* coefficients and associated *p* values. A linear regression analysis was performed to rule out any possible interaction between sex and dairy product intake in relation to bone properties, by including sex × dairy intake as an independent variable. Additionally, a sensitivity analysis was completed in which other items from the FFQ (e.g., sweets, root vegetables) were substituted for dairy in the regression models in order to assess systematic bias between reported dietary habits and bone properties. One-way analysis of covariance (ANCOVA) adjusted for sex, average portion size, dietary protein content, cross-sectional muscle area measured by pQCT, smoking, and physical activity was used to investigate differences in the effects of different dairy product types on bone properties. Two-sided *p* values < 0.05 were considered to indicate significant differences for all analyses. A Bonferroni correction was also carried out in relation to the regression analyses between dairy consumption and bone parameters in which the significance threshold was divided by the number of analyses (*n* = 13, 0.05/13 = 0.0038). Statistical analyses were performed using the SPSS software (version 23; IBM, Armonk, NY, USA). Normal distribution was assumed for all bone measurements.

## Results

### Study cohort

The characteristics of the study cohort are presented in Table 1. Men reported a higher milk only consumption than women (*p* < 0.001) and a lower fermented dairy consumption (*p* < 0.05) than women. Smoking habits and total physical activity did not differ significantly between men and women. Values for all measured bone parameters were greater among men than among women (*p* < 0.001 for all). The combined sex × dairy intake variable was not associated significantly with any bone parameter, indicating the absence of an interaction effect. Thus, data from men and women were combined in all further analyses.

**Table 1:** Characteristics of the study cohort

	All (*n* = 2040)	Women (*n* = 1000)	Men (*n* = 1040)
Age (years)	70	70	70
Height (m)	1.70 ± 0.09	1.63 ± 0.06	1.77 ± 0.06**
Weight (kg)	77.1 ± 14.5	70.2 ± 12.9	83.7 ± 12.8**
BMI (kg/m^2^)	26.6 ± 4.2	26.3 ± 4.6	26.8 ± 3.7*
Smoker, yes (%)	6.0	7.0	5.2
Dairy intake			
Total amount of dairy consumed (dl)	3.3 ± 1.9	3.0 ± 1.7	3.6 ± 2.2**
MG (%)	10.0	7.7	12.1**
FDG (%)	13.3	15.2	11.5*
ADG (%)	66.8	68.3	65.3
NDG (%)	3.8	3.4	4.3
Accelerometer measurements			
LPA (min/day)	201.7 ± 52.1	212.0 ± 52.4	191.81 ± 49.9**
MVPA (min/day)	32.7 ± 26.1	30.4 ± 23.2	35.0 ± 28.6**
Total activity/total wear time (counts/min)	921.6 ± 340.9	914.9 ± 304.6	928.1 ± 372.6
pQCT measurements			
Tibia			
Trabecular CSA^5a^ (mm^2^)	537.6 ± 91.6	481.3 ± 69.6	591.7 ± 76.5**
Trabecular BMD^6a^ (mg/cm^3^)	218.6 ± 41.0	203.1 ± 40.0	233.6 ± 36.2**
Cortical CSA^b^ (mm^2^)	296.7 ± 69.9	243.1 ± 40.6	348.2 ± 50.9**
Cortical BMD^b^ (mg/cm^3^)	1088.8 ± 38.7	1077.8 ± 40.4	1099.3 ± 33.7**
Cortical thickness^b^ (mm)	3.7 ± 0.8	3.3 ± 0.7	4.2 ± 0.7**
Radius			
Trabecular CSA^a^ (mm^2^)	199.7 ± 40.8	171.3 ± 24.2	226.9 ± 34.5**
Trabecular vBMD^a^ (mg/cm^3^)	181.4 ± 43.9	158.2 ± 37.7	203.6 ± 37.5**
Cortical CSA^b^ (mm^2^)	82.5 ± 24.8	62.3 ± 13.1	101.8 ± 16.8**
Cortical vBMD^b^ (mg/cm^3^)	1104.9 ± 49.3	1090.7 ± 50.1	1118.5 ± 44.6**
Cortical thickness^b^ (mm)	2.2 ± 0.6	1.8 ± 0.4	2.6 ± 0.4**
DXA measurements			
Femoral neck aBMD (mg/cm^2^)	871.6 ± 133.8	816.7 ± 112.8	924 ± 131.2**
L1–L4 vertabrae aBMD (mg/cm^2^)	1151.4 ± 214.3	1052.9 ± 180.3	1246.3 ± 201.3**
Radius aBMD^c^ (mg/cm^2^)	847.6 ± 160.3	721.7 ± 111.6	967.7 ± 93.8**

Values are presented as means ± Standard deviations, except for smoking, intake of milk, and intake of fermented dairy products which are presented as percentages

*LPA* light physical activity, *MVPA* moderate and vigorous physical activity, *vBMD* volumetric bone mineral density, *aBMD* areal BMD, *CSA* cross-sectional area, *MG* milk group (milk only, no fermented dairy products), *FDG* fermented dairy group (fermented dairy products only, no milk), *ADG* all dairy group (consumes both milk and fermented dairy products), *NDG* no dairy group (consumes neither milk nor fermented dairy products)

^a^Measured at the 4% site

^b^Measured at the 66% site

^c^Measured at the 33% site

*p ≤ 0.05 significant difference between men and women

**p ≤ 0.001 significant difference between men and women

### Total dairy product intake and bone properties

**Fig. 2:**
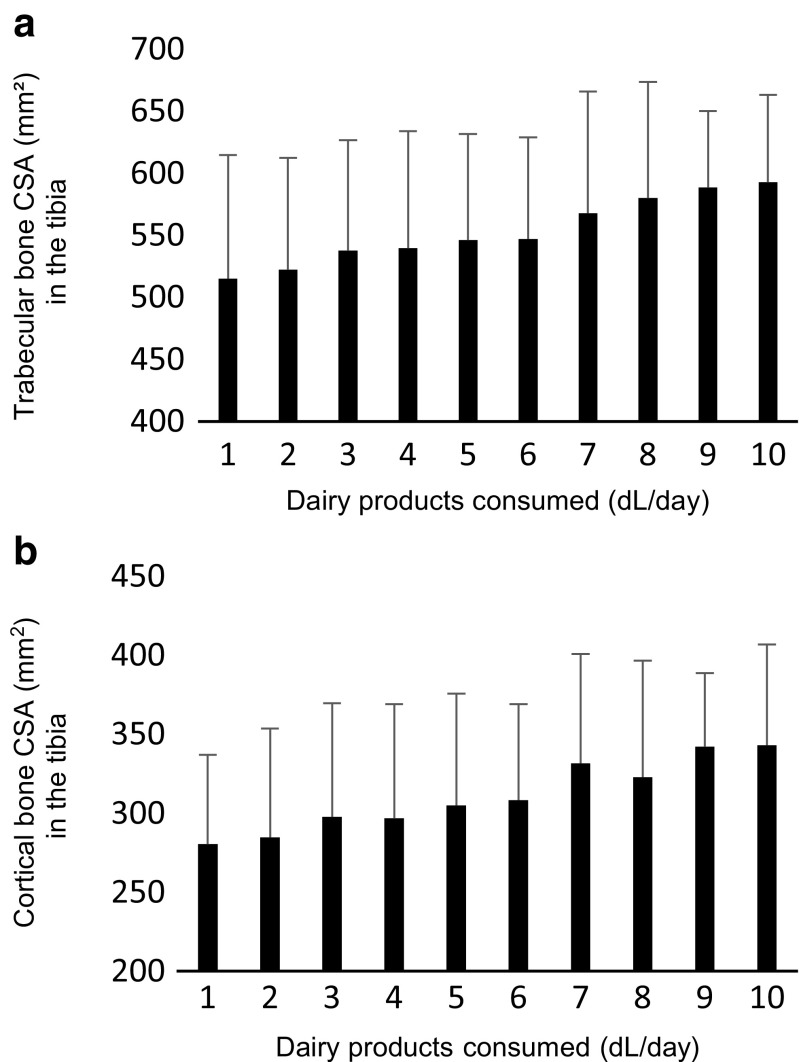
The linear relationship between dairy product consumption and **a** trabecular or **b** cortical cross-sectional area (CSA) in the tibia. Error bars represent standard deviations

Descriptive analysis indicated a positive linear association between the amount of dairy product consumed and CSA in the tibia as shown in Fig. 2. The associations between total dairy product intake and bone properties in two models are presented in Table 2. In the first model, adjusted for sex only, the amount of dairy products consumed showed weak positive associations with trabecular and cortical CSAs in the tibia. No association was observed between the amount of dairy products consumed and trabecular vBMD, cortical vBMD, or cortical thickness in the tibia or any property in the radius as assessed by pQCT. The observed associations remained in the fully adjusted model for trabecular CSA (*β* = 0.049, *p* = 0.01 representing 2.296 (95% CI 0.552–4.039) mm^2^ increase per dl dairy product) and cortical CSA (*β* = 0.049, *p* = 0.001, representing 1.757 (95% CI 0.683–2.830 mm^2^) increase per dl dairy product). Furthermore, femoral neck aBMD and L1–L4 vertebrae aBMD as assessed by DXA were not significantly associated with diary consumption in any of the models. However, radius BMD as measured at the 33% distal site showed a weak but significant association to dairy consumption (*β* = 0.039, *p* = 0.01, representing 3.231 (95% CI 0.764–5.698) mg/cm^2^ increase per dl dairy product). After Bonferroni corrections of multiple comparisons (*n* = 13), only cortical CSA area remained significantly associated to dairy consumption. Sex, followed by cross-sectional muscle area, showed the strongest associations with all bone measurements (data not presented). Additionally, none of the other FFQ items included in the sensitivity analysis was significantly correlated to any of the investigated bone parameters in the regression model. Finally, inclusion of cheese consumption in the adjusted regression model did not significantly alter the results (data not presented).

**Table 2:** Results of linear regression models examining self-reported dairy product consumption as an independent predictor of the investigated bone properties

Measurement site	Model 1		Model 2		Unstandardized	
	Stand. *β*	*p*	Stand. *β*	*p*	*β*	95% CI
pQCT						
Tibia						
Trabecular CSA (mm^2^)	0.041	**0.024**	0.049	**0.010**	2.296	0.552–4.039
Trabecular vBMD (mg/cm^3^)	0.007	0.733	0.013	0.575	0.263	− 0.670–1.260
Cortical CSA (mm^2^)	0.043	**0.005**	0.049	**0.001**	1.757	0.683–2.830
Cortical vBMD (mg/cm^3^)	− 0.012	0.600	0.014	0.555	0.283	− 0.353–1.221
Cortical thickness (mm)	0.031	0.099	0.037	0.057	0.015	− 0.000–0.031
Radius						
Trabecular CSA (mm^2^)	0.001	0.935	0.013	0.453	0.282	− 0.455–1.019
Trabecular vBMD (mg/cm^3^)	0.018	0.357	0.028	0.177	0.649	− 0.294–1592
Cortical CSA (mm^2^)	0.015	0.273	0.025	0.087	0.324	− 0.047–0.695
Cortical vBMD (mg/cm^3^)	− 0.01	0.657	0.014	0.545	0.374	− 0.837–1.585
Cortical thickness (mm)	0.011	0.536	0.028	0.128	0.008	− 0.002–0.019
iDXA						
Femoral neck aBMD (mg/cm^2^)	0.024	0.262	0.027	0.237	1.844	− 1.217–4-905
L1–L4 vertabrae aBMD (mg/cm^2^)	− 0.007	0.742	− 0.008	0.691	− 0.925	− 5.554–3.684
Radius aBMD (mg/cm^2^)	0.028	0.054	0.039	**0.010**	3.231	0.764–5.698

Model 1 is adjusted for sex. Model 2 is adjusted for sex, average portion size, dietary protein content, cross-sectional muscle area measured by pQCT, smoking, and physical activity. Values are presented as standardized β coefficients in models 1 and 2. The unstandardized β coefficients with the 95% confidence interval from model 2 is also given, quantified to 1 dl of dairy product consumed. Values in bold font indicate *p* < 0.05

*vBMD* volumetric bone mineral density, *aBMD* areal BMD, *CSA* cross-sectional area, *CI* confidence interval

### Type of dairy product and bone properties

Covariate-adjusted means (adjusted for sex, muscle area, average portion size, dietary protein content, smoking, and physical activity) for bone parameters stratified by type of dairy consumption are given in Table 3. Statistical differences between dairy product groups were investigated with ANCOVA using the same adjusting variables as stated above. None of the dairy product groups were significantly different from the other in any of the investigated bone parameters.

**Table 3:** Adjusted means of bone properties categorized by type of dairy product consumed

	Dairy group			
Measurement site	MG (*n* = 203)	FDG (*n* = 274)	ADG (*n* = 1373)	NDG (*n* = 79)
pQCT				
Tibia				
Trabecular CSA (mm^2^)	535 ± 4	545 ± 4	536 ± 2	535 ± 9
Trabecular vBMD (mg/cm^3^)	219 ± 3	220 ± 2	218 ± 1	213 ± 5
Cortical CSA (mm^2^)	293 ± 3	295 ± 3	297 ± 1	293 ± 5
Cortical vBMD (mg/cm^3^)	1088 ± 3	1090 ± 3	1089 ± 1	1077 ± 4
Cortical thickness (mm)	3.7 ± 0	3.7 ± 0	3.7 ± 0	3.7 ± 0
Radius				
Trabecular CSA (mm^2^)	197 ± 2	201 ± 2	198 ± 1	201 ± 4
Trabecular vBMD (mg/cm^3^)	180 ± 3	182 ± 3	181 ± 1	180 ± 4
Cortical CSA (mm^2^)	81 ± 1	82 ± 1	82 ± 0	81 ± 2
Cortical vBMD (mg/cm^3^)	1103 ± 3	1108 ± 3	1105 ± 1	1096 ± 6
Cortical thickness (mm)	2.1 ± 0	2.1 ± 0	2.1 ± 0	2.1 ± 0
iDXA				
Femoral neck aBMD (mg/cm^2^)	882 ± 9	873 ± 8	871 ± 4	852 ± 14
L1–L4 vertabrae aBMD (mg/cm^2^)	1162 ± 14	1165 ± 13	1142 ± 5	1140 ± 22
Radius aBMD (mg/cm^2^)	838 ± 7	841 ± 7	845 ± 3	840 ± 12

Values are presented as adjusted means ± standard errors (adjusted for sex, average portion size, dietary protein content, cross-sectional muscle area measured by pQCT, smoking, and physical activity)

*MG* milk group (milk only, no fermented dairy products), *FDG* fermented dairy group (fermented dairy products only, no milk), *ADG* all dairy group (milk and fermented dairy products), *NDG* no dairy group (no milk or fermented dairy products), *vBMD* volumetric bone mineral density, *CSA* cross-sectional area, *aBMD* areal bone mineral density

## Discussion

In the present investigation of 2040 elderly men and women, we explored the relationships between dairy product consumption and bone properties, as measured by pQCT and DXA. Our findings suggest the existence of weak but significant positive associations of dairy product intake with the properties of predominantly bone area, expressed as higher cortical and trabecular CSAs in the tibia. We found no indication of a negative effect of dairy product consumption on any investigated bone measurement.

The mineralization of the skeleton undeniably decreases with age [22], and low BMD has been linked to an increased risk of fracture in elderly men and women [23–29], as well as in very elderly women [30, 31]. Calcium supplementation has been shown to reduce the loss of bone [32], and increased consumption of calcium-rich dairy products (e.g., milk) has been found to provide an adequate source of dietary calcium, comparable to the results of other calcium supplementation strategies [33]. Even so, the association between dairy product consumption and bone health in elderly adults has not been firmly established [4, 7–9, 34], and further investigation is warranted. Although the majority of studies point to a positive relationship, some findings suggest that dairy product consumption has detrimental effects. Specifically, a recent study by Michaelsson et al. [10] showed that milk intake was associated with higher mortality and fracture rates, which led the authors to question the recommendation of milk intake to counter fragility fractures. Although we did not investigate the relationship between dairy product consumption and fracture per se, our finding of no negative association with bone parameters suggest that dairy consumption is not detrimental for bone health, at least in our population. Furthermore, other authors have reported the lack of association of milk intake with mortality [35, 36] and fracture risk [7, 8, 37], further challenging the findings relating milk to unfavorable health effects. In the same study, Michaelsson et al. [10] found that the consumption of fermented dairy products was associated with lower rates of fractures and mortality. We found no support for this discrepancy in the present study, as the consumption of milk and fermented products did not have significantly different impacts on the investigated bone properties.

In the present study, we used pQCT together with the more commonly employed DXA, to evaluate different aspects of the bone [24, 25, 28, 30]. pQCT enables measurement of cortical and trabecular bone, as well as CSAs, thereby enabling more in-depth examination of bone properties. To our knowledge, our study is the first to investigate the associations between dairy product intake and bone properties measured by pQCT in both elderly men and women. Given the demonstrated association with CSA of the tibia (which cannot be measured with DXA), the inclusion of pQCT data was relevant. Overall, dairy intake showed weak positive associations with bone properties at some sites investigated. Furthermore, based on confidence limits, the associations were similar for weight-bearing bone and non-weight bearing bone, similar for peripheral and axial bone sites, and similar for bone properties measured by pQCT and DXA. In summary, none of the associations indicate a negative influence of higher milk intake on different estimates of bone strength in this cohort of 70-year-old men and women.

The present population-based investigation has strengths and limitations that should be considered when interpreting the results. Firstly, due to the cross-sectional design, any claim of a causal relationship cannot be established. Information on participants’ use of calcium and vitamin D supplements was not acquired and could thus not be added to the adjustments in the regression analyses. The assessment of participants’ dairy product intake was based only on FFQ responses about participants’ usual food habits, which may have changed during their lifetimes. Also, although the questions on dairy consumption have not been internally validated, the use of quantified, self-reported dairy intake have previously been validated by others against 7-day weighted intake records [38] showing close to identical results. In addition, FFQ responses are subject to systematic and random errors, which may have affected the results [39], although our sensitivity analysis did not indicate a systematic error which, together with the use of meal size and protein proportion as adjusting variables, gives additional strength to our findings. Furthermore, the chosen method was logistically preferable to the use of interview-based assessments, due to the large number of participants [40]. The use of accelerometers to objectively measure physical activity allowed data collection for only 1 week, although this approach may be preferable to the assessment of self-recalled physical activity, considering the effects of recall bias [16]. Although we had access to a large number of covariates, there is likely residual confounding in the associations found. Lastly, the participants were all 70 years of age and lived in northern Sweden; thus, they represent a well-defined homogenous reference group, but the findings are not necessarily generalizable to younger or older individuals. Future studies should investigate whether similar associations between bone health and dairy product consumption to that found in this study can be detected in other age groups.

In conclusion, this study revealed no negative association between dairy product consumption and bone health. Instead, we found a weak positive association between dairy product consumption and some of the bone sites, regardless of the type of dairy product consumed. Our findings do not suggest that higher dairy product intake has negative effects on various bone properties in 70-year-old men and women.
